# Responses of hydrolytic enzyme activities in saline-alkaline soil to mixed inorganic and organic nitrogen addition

**DOI:** 10.1038/s41598-018-22813-9

**Published:** 2018-03-14

**Authors:** Baoku Shi, Junmei Zhang, Chengliang Wang, Jianying Ma, Wei Sun

**Affiliations:** 1 Key Laboratory for Vegetation Ecology, Ministry of Education, Institute of Grassland Science, Northeast Normal University, Changchun, 130024 China; 2 State Key Laboratory of Desert and Oasis Ecology, Xinjiang Institute of Ecology and Geography, Chinese Academy of Sciences, Urumqi, 830011 China

## Abstract

The effects of manipulating nitrogen (N) deposition, with the use of a single form of N, on soil enzyme activities have been extensively studied. However, the impacts varying the N type (organic vs. inorganic) on soil hydrolytic enzyme activities have been less studied. We performed a 60 day incubation experiment using saline-alkaline soil. The objectives were to explore how the microbial biomass and enzyme activities respond to a mixed N addition at different inorganic to organic N ratios. The experimental design was full factorial, with two rates of N addition (10 g N m^−2^ and 20 g N m^−2^) and four ratios of N addition (inorganic N:organic N = 10:0, 7:3, 3:7, 1:9). The results showed that N addition stimulated enzyme activities involved in C, N and P cycling. Enzyme activities under mixed N addition increased compared to those under single inorganic N addition in most cases. The inorganic to organic N ratios interacted with the N addition rate to affect the enzyme activities. Our results suggest that various N fertilizers, which have different inorganic to organic N ratios, should be applied when evaluating the effects of atmospheric N deposition on the soil microbial enzyme activities and ecosystem structure and function.

## Introduction

Soil hydrolytic enzymes mediate soil organic matter decomposition, transformation, and mineralization and play a pivotal role in decomposition and nutrient cycling^1–3^. Enzyme activities depend on a series of abiotic factors, such as the soil temperature, soil water content and pH^4,5^. The main biotic processes that influence enzyme activities are enzyme synthesis and secretion, which are normally governed by local substrate concentrations and nutrient availability^6^. Nitrogen (N) is the major limiting nutrient in temperate terrestrial ecosystems^7^. The elevated atmospheric N deposition due to human activities have increased N availability, alleviated soil N limitation, further altered soil microbial biomass and enzyme activities^8^. These changes will have important consequences for ecosystem functions such as organic matter decomposition, nutrient cycling and plant-microbe interactions^6^. Thus, understanding how soil enzyme activities respond to simulated N deposition is important.

The effects of increased N deposition on plant community composition, biodiversity and aboveground productivity have been extensively studied^9,10^. N enrichment can increase plant aboveground productivity and lead to biodiversity loss^9,10^. Although the effects of increased N deposition on soil enzyme activities have received considerable research attention, N enrichment has been shown to have complicated and variable effects on soil enzyme activities. In some ecosystems, β-glucosidase^11^ or leucine aminopeptidase^12^ increase with N amendment, but other studies have found that N addition significantly decreases β-glucosidase and leucine aminopeptidase activities^13^ or leads to non-significant changes in β-glucosidase^5^. The observed inconsistent results in the literature may be attributed to differences in vegetation and soil types, and the form and level of N fertilization^2^.

Most studies on the effects of N deposition on soil enzyme activities use a single form of N. However, natural atmospheric N deposition includes both inorganic N (e.g., ammonium and nitrate) and organic N (e.g., urea, peptides and dissolved free amino acids)^14^. Thus, simulated N deposition experiments using a single form of N cannot mimic the real components of atmospheric nitrogen deposition. Previous research has reported that mixed N fertilization stimulated soil enzyme activities, with more pronounced effects than single inorganic N (NH_4_NO_3_) fertilization^15–17^. Compared to inorganic N, organic N (such as glycine) can be rapidly assimilated by microorganisms and has more significant promoting effects on microbial activities^18^. However, as far as we know, all of the published studies on the responses of hydrolytic enzyme activities to mixed N addition at different inorganic to organic N ratios have focused on forest ecosystems with high soil acidity^19^, and no information is available for saline-alkaline grassland. Soil enzyme activities are sensitive to variation in soil pH^20^. Chronic nitrogen deposition not only alters soil N availability, but also can lead to soil acidification^16,21^. Soil acidification may reduce microbial biomass and inhibit enzyme activities as a result of the direct effects of the decreased pH on microbial physiology, biologically toxic effects of increased Al^3+^ ^21^, or base cation limitation^22^. For saline-alkaline soil (normally pH value > 8), N addition induces a decrease in soil pH and can lead to positive effects on microorganisms. However, this hypothesis has not yet been tested.

The N addition rate has been documented to influence soil microbial biomass and enzyme activities^5,11,14–17,23^. The high nitrogen addition rate associated with NO_3_^−^ accumulation had a deleterious effect on microbial groups, and thus, decreased soil microbial biomass and hydrolytic enzyme activities^5,23^. As reported in a Mediterranean forest soil, the effect of N fertilization on soil enzyme activities (one oxidative enzyme and eight hydrolytic enzymes) was nonlinear, and depended on the microbial community dynamics, the community’s ability to adapt given the time scale of the process, and the N supply rate^24^. Nevertheless, it is not clear whether the ratio of inorganic to organic N and the N addition rate interactively or additively influence microbial biomass and enzyme activities when they are taken into consideration together.

The Songnen Plain is located in Northeast China and consists of a natural vegetation of meadow steppe dominated by *Leymus chinensis*. The Songnen Plain is at a low elevation (<100 m) and is surrounded by mountains with very poor drainage. Its evaporative demands are almost three times that of the annual precipitation, which brings minerals dissolved in groundwater from deep soil to the surface^25^. Thus, the topographical features and climatic conditions of this area lead to a soil alkalization and salinization. The vast alkalinized-salinized grassland covers 23 925.79 km^2^, and accounts for approximately two-thirds of the meadow steppe area of the Songnen Plain^26^. Soil samples were collected from this alkalinized-salinized grassland and subjected to N addition treatments, which provided an opportunity to explore the response of hydrolytic enzyme activities in saline-alkaline soil to mixed inorganic and organic nitrogen addition. We investigated the microbial biomass C (MBC) and seven hydrolytic enzyme activities involved in decomposition or cycling of organic C, N or phosphorus (P) under simulated N deposition using four different inorganic to organic N ratios and two rates of N addition during a 60 day incubation period. Our specific objectives were to (1) explore how microbial biomass and enzyme activities respond to a mixed N addition at different inorganic to organic N ratios in saline-alkaline soil and (2) determine if this response depends on the N addition rate.

## Results

### Soil enzyme activities

**Figure 1:**
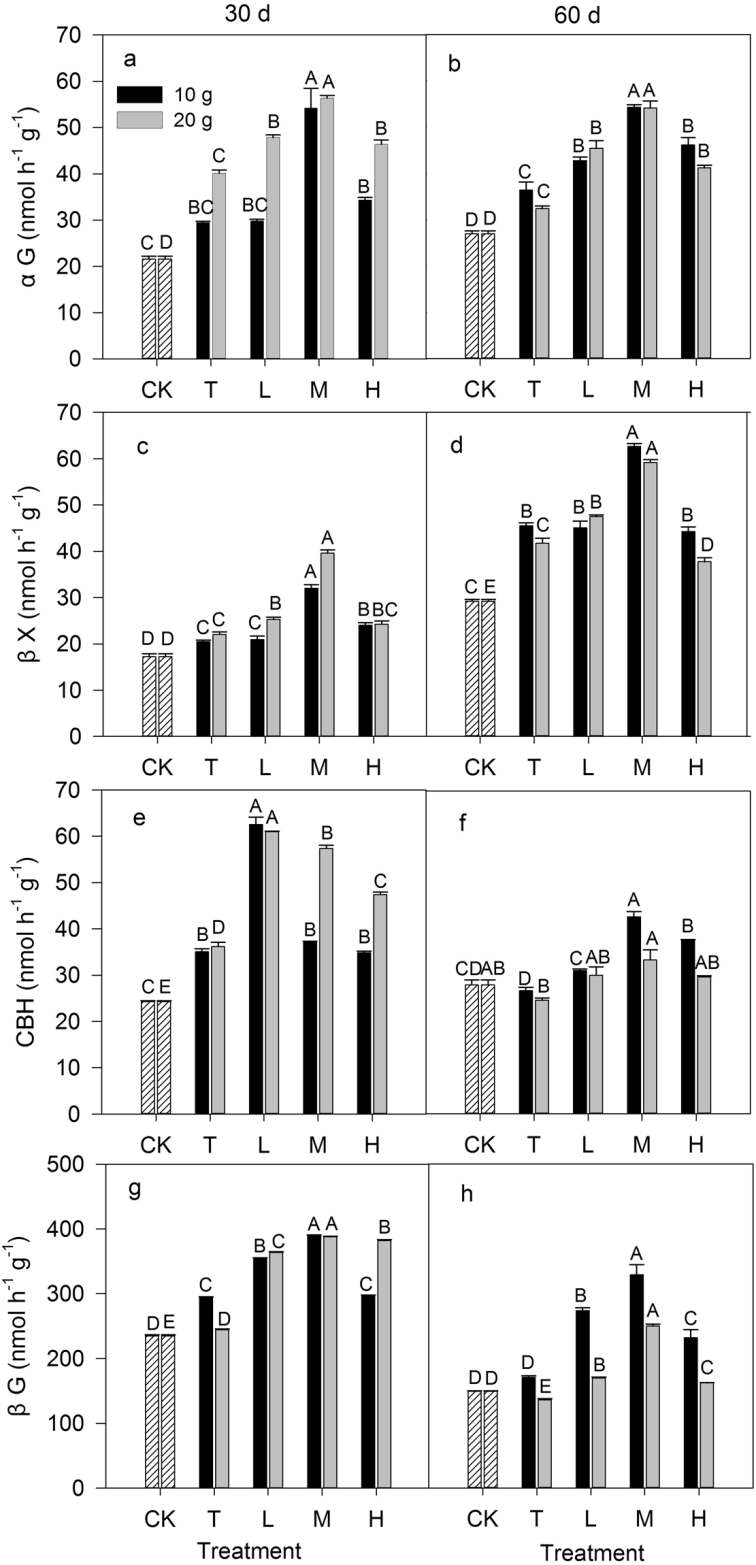
Response of soil enzyme activities involved in C cycling to different inorganic to organic N ratios, different N rate (10 g N m^−2^ and 20 g N m^−2^) and different incubation time (30 day and 60 day). CK: control treatment; T: total inorganic N addition; L: low organic N addition; M: medium organic N addition; H: high organic N addition. Different letters indicate significant differences among different inorganic to organic N ratios (*P* < 0.05).

Compared to the CK treatment, N addition stimulated the enzyme activities involved in C cycling, such as αG, βX, CBH, and βG in most cases during the 60-day incubation period (Fig. 1). N addition induced stimulatory effects on enzyme activities involved in C cycling, and these effects differed among the different N ratio treatments. The enzyme activities under the mixed N addition (L, M and H) treatments were, on average, higher than those under the T treatments. Within the mixed N addition treatments, αG, βX and βG under the M treatments showed the highest activities of all of the N addition treatments; but for CBH, the highest activity was observed under the L treatment at day 30. The N addition rate interacted with the incubation time and affected enzyme activities (*P* < 0.001, Table 1). The activities of αG, βX, CBH, and βG under the 20 g N m^−2^ N addition treatment were, on average, 29%, 14%, 19% and 3%, respectively, higher than those of the 10 g N m^−2^ treatment at day 30 and were, on average, 4%, 6%, 15% and 29%, respectively, lower than those of the 10 g N m^−2^ treatment at day 60. In addition, there was a significant effect of the interaction among the N ratio, N addition rate, and incubation time on the enzyme activities involved in C cycling (Table 1).

**Table 1:** Results (*P* values) of three-way ANOVA on the effects of the N ratio, N addition rate, incubation time, and their interactions on soil enzyme activities (nmol h^−1^ g^−1^), microbial biomass C (MBC, mg kg^−1^) and pH.

Factors	αG	βX	CBH	βG	LAP	NAG	ALP	MBC	pH
N ratio	<0.001	<0.001	<0.001	<0.001	<0.001	<0.001	<0.001	<0.001	<0.001
N rate	<0.001	0.421	0.002	<0.001	<0.001	<0.001	<0.001	<0.001	<0.001
Time	<0.001	<0.001	<0.001	<0.001	<0.001	<0.001	0.984	<0.001	<0.001
N ratio × N rate	<0.001	<0.001	<0.001	<0.001	<0.001	<0.001	<0.001	<0.001	<0.001
N ratio × Time	0.005	<0.001	<0.001	<0.001	<0.001	<0.001	<0.001	<0.001	<0.001
N rate × Time	<0.001	<0.001	<0.001	<0.001	<0.001	<0.001	<0.001	<0.001	<0.001
N ratio × N rate × Time	0.001	0.001	<0.001	<0.001	<0.001	<0.001	<0.001	0.994	<0.001

**Figure 2:**
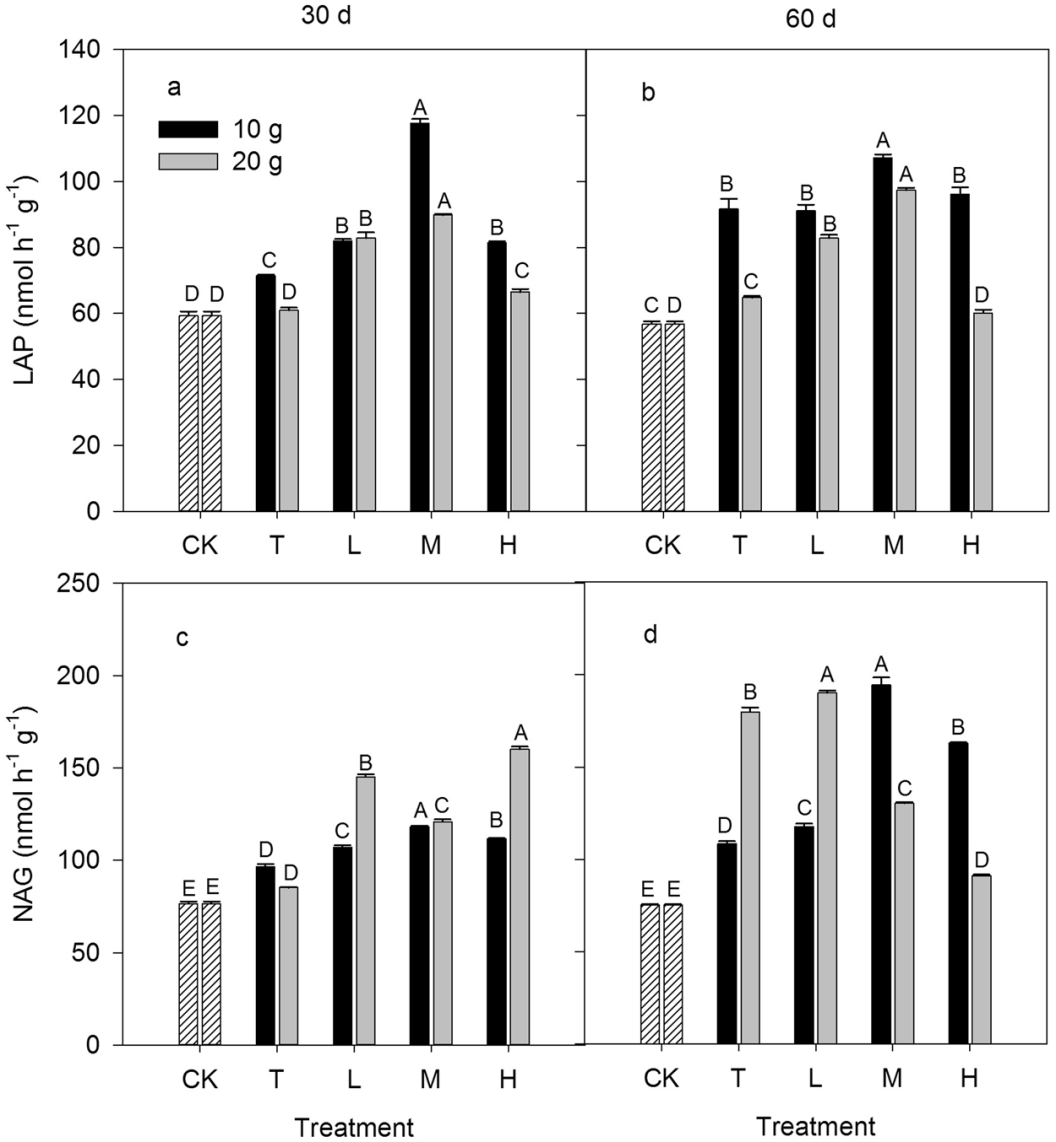
Response of soil enzyme activities involved in N cycling to different inorganic to organic N ratios, different N rate (10 g N m^−2^ and 20 g N m^−2^) and different incubation time (30 day and 60 day). CK: control treatment; T: total inorganic N addition; L: low organic N addition; M: medium organic N addition; H: high organic N addition. Different letters indicate significant differences among different inorganic to organic N ratios (*P* < 0.05).

The ratio of inorganic to organic N significantly affected soil enzyme activities (LAP and NAG) involved in N cycling (*P* < 0.05, Fig. 2). The LAP and NAG activities under N addition treatments were higher than those of the control in most cases during the 60 day incubation period. The highest LAP activities were detected under the M treatments. The NAG activities under the M treatments were the highest and were the lowest under the T treatments in all of 10 g N m^−2^ N addition treatments regardless of the incubation time. The NAG activities under the H and L treatments of all of the 20 g N m^−2^ N addition treatments were the highest at days 30 and 60, respectively.

**Figure 3:**
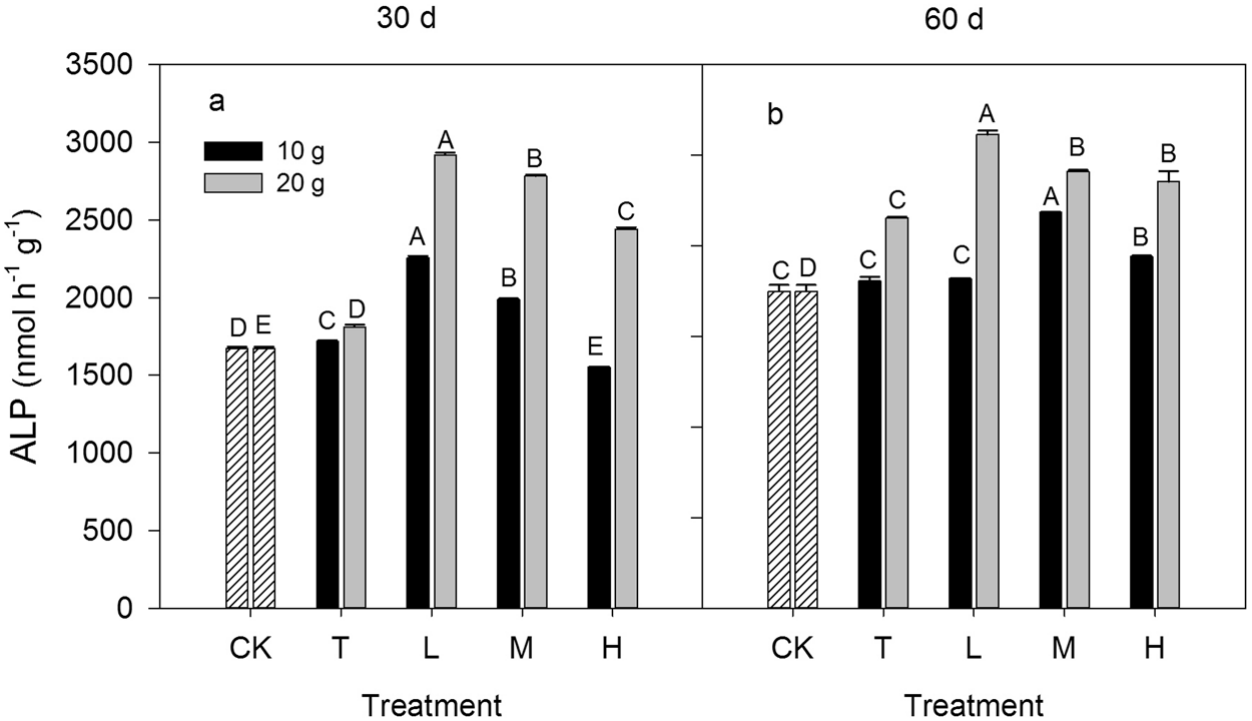
Response of soil enzyme activities involved in P cycling to different inorganic to organic N ratios, different N rate (10 g N m^−2^ and 20 g N m^−2^) and different incubation time (30 day and 60 day). CK: control treatment; T: total inorganic N addition; L: low organic N addition; M: medium organic N addition; H: high organic N addition. Different letters indicate significant differences among different inorganic to organic N ratios (*P* < 0.05).

**Figure 4:**
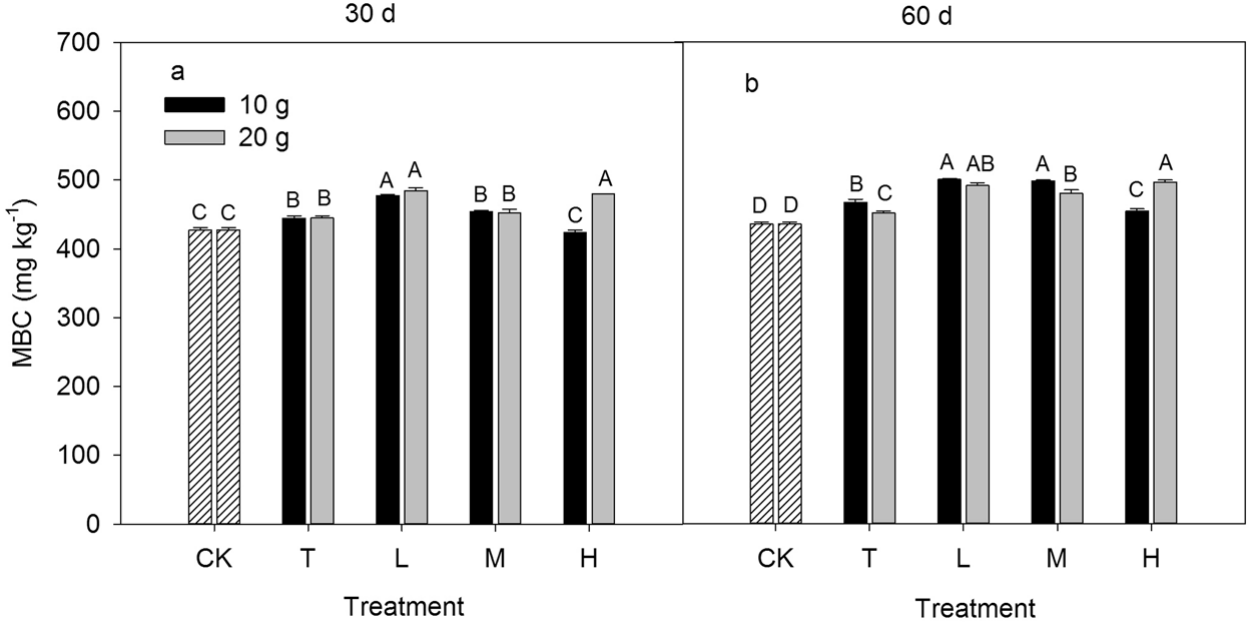
Response of soil microbial biomass C (MBC) to different inorganic to organic N ratios, different N rate (10 g N m^−2^ and 20 g N m^−2^) and different incubation time (30 day and 60 day). CK: control treatment; T: total inorganic N addition; L: low organic N addition; M: medium organic N addition; H: high organic N addition. Different letters indicate significant differences among different inorganic to organic N ratios (*P* < 0.05).

Similar to the soil enzyme activities involved in C and N cycling, the activities of alkaline phosphatase (ALP) were significantly affected by the ratio of inorganic to organic N (*P* < 0.05, Fig. 3). N addition stimulated ALP activity except for the 10 g N m^−2^ H treatment at day 30. The ALP activities under the 20 g N m^−2^ N addition treatments at day 30 were higher than the control treatment and the stimulating effects on ALP activity by the mixed N addition (L, M and H) treatments were higher than those of the T treatments. The ALP activity under the 10 g N m^−2^ N addition treatments at day 60 was increased in the order of CK = T = L < H < M, while was increased in the order of CK < T < H = M < L under the 20 g N m^−2^ N addition treatments. The ALP activities under the 20 g N m^−2^ N addition treatments were significantly higher than under the 10 g N m^−2^ N addition treatments during the 60 day incubation period (*P* < 0.05). Moreover, the enzyme activities normalized to the soil dry mass or MBC displayed similar variation patterns of activity across the different N addition rates and ratios (Fig. 4; Figs S1–3).

### Soil microbial biomass

**Figure 5:**
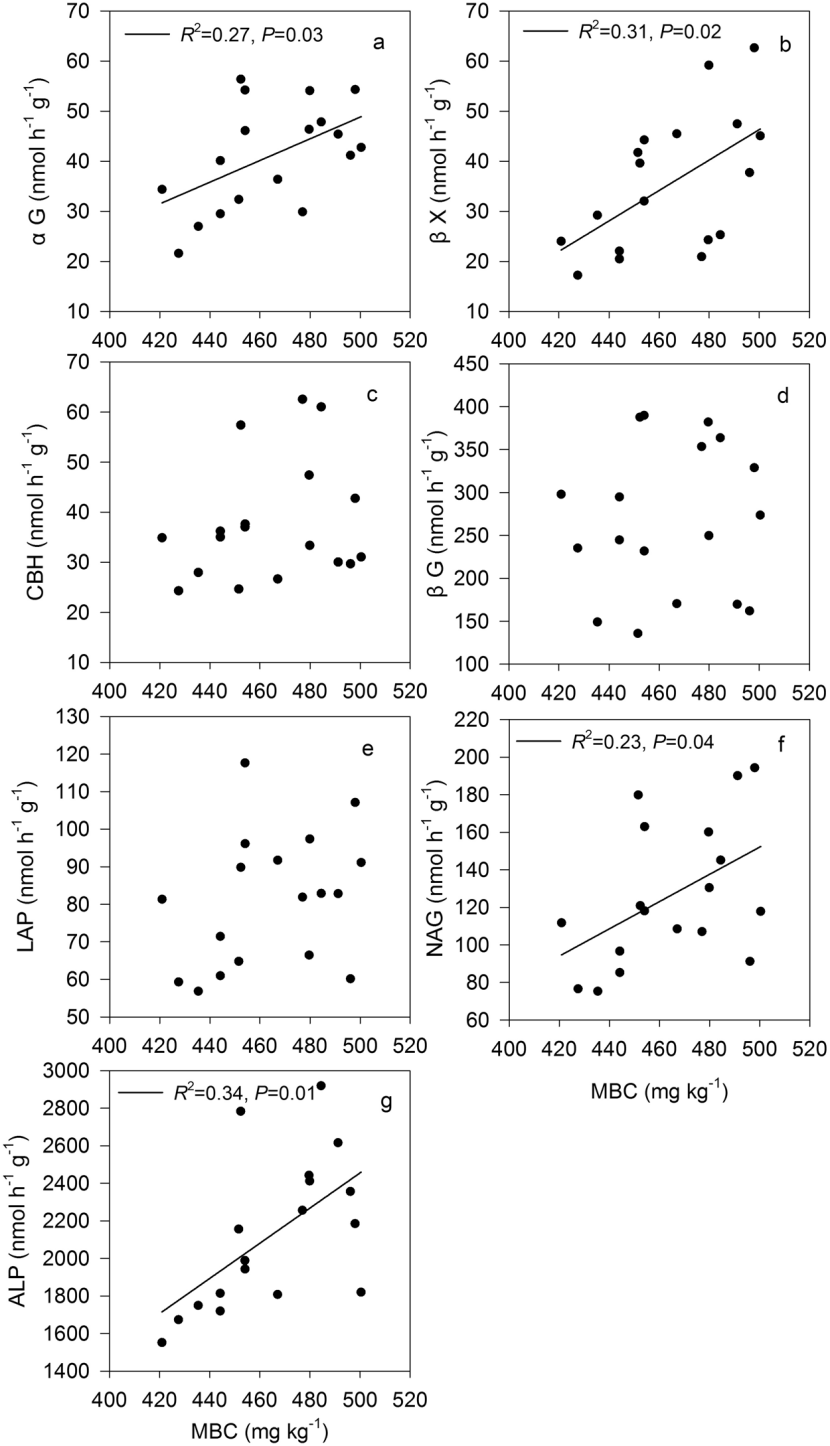
Dependence of soil enzyme activities on soil microbial biomass C (MBC) across different N addition rates and ratios.

MBC differed significantly among the N ratio treatments (*P* < 0.05, Fig. 4). The MBC of the 10 g N m^−2^ L and M treatments at day 30 were significantly higher than that of the control treatment (*P* < 0.05), whereas no significant difference was detected between the control and T and H treatments (*P* = 0.09; 0.77). The MBC under the N addition rate of 10 g N m^−2^ at day 60 was increased in the order of CK < H = T < M = L. The MBC of the control treatment was similar to that of the T treatment at 20 g N m^−2^ regardless of the incubation time (*P* = 0.21; 0.30). However, there were significant increases of MBC in all of the mixed N addition treatments at 20 g N m^−2^ during the 60-day incubation period (*P* < 0.05). The MBC had a significant positive correlation with αG, βX, NAG and ALP (*P* < 0.05, Fig. 5), while we did not find significant relationships between the MBC and CBH, βG and LAP (*P* = 0.34; 0.74; 0.20).

### Soil pH

**Figure 6:**
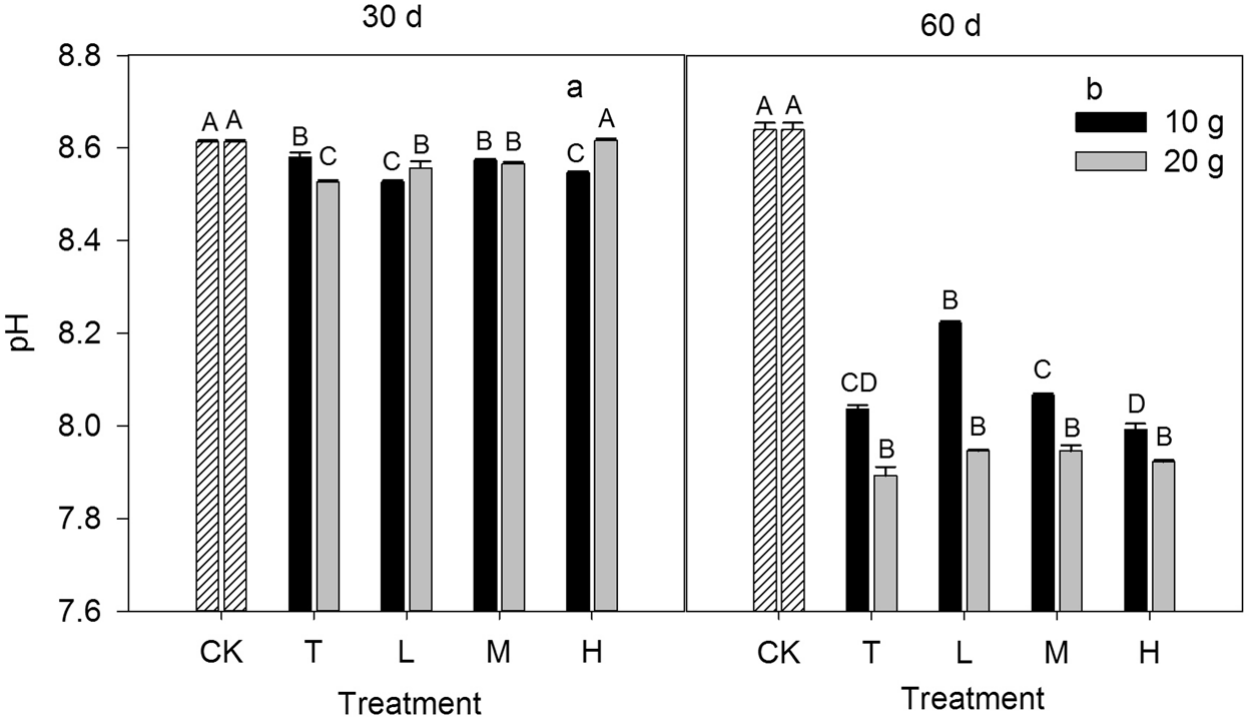
Response of soil pH to different inorganic to organic N ratios, different N rates (10 g N m^−2^ and 20 g N m^−2^) and different incubation times (30 days and 60 days). CK: control treatment; T: total inorganic N addition; L: low organic N addition; M: medium organic N addition; H: high organic N addition. Different letters indicate significant differences among different inorganic to organic N ratios (*P* < 0.05).

**Figure 7:**
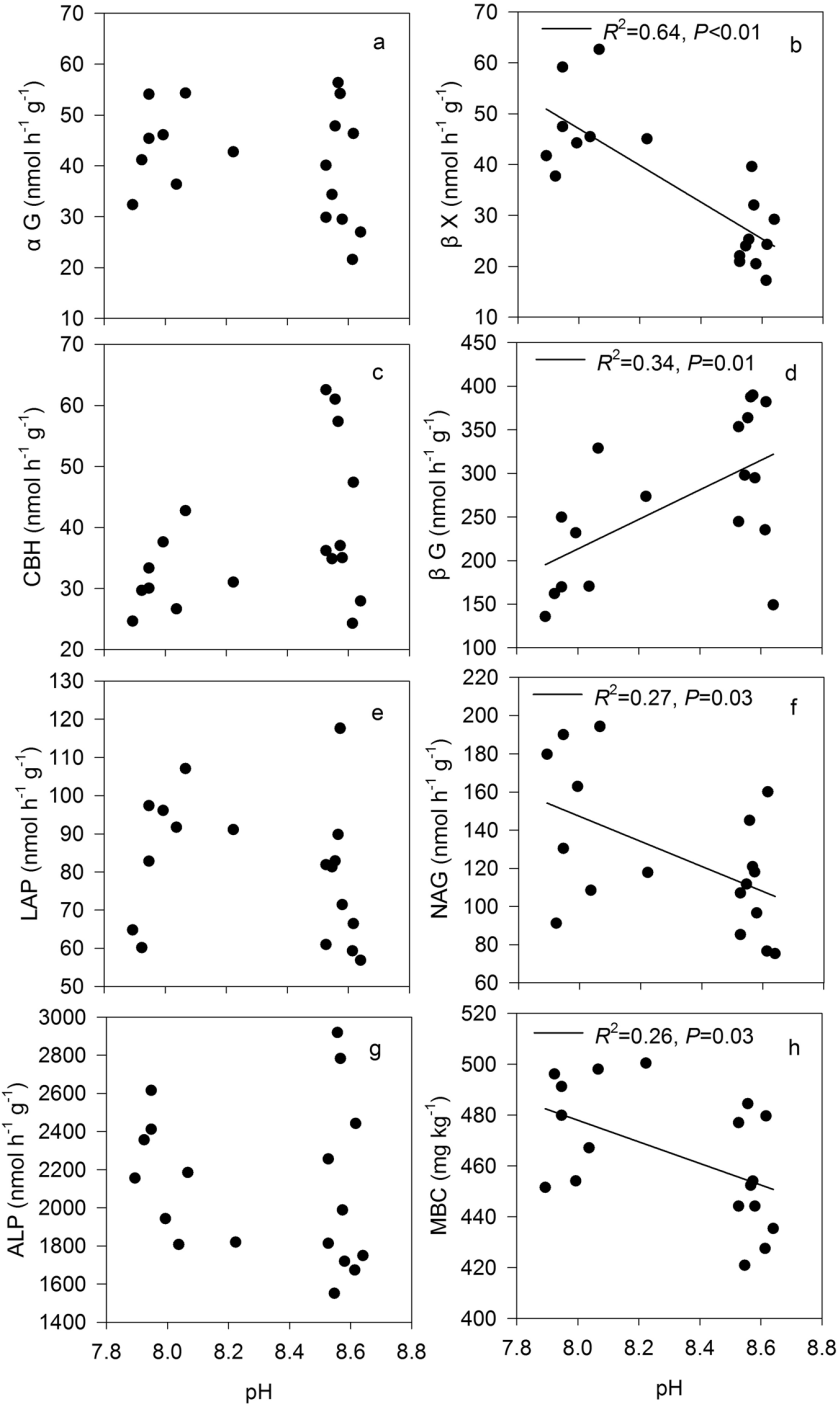
Relationships between soil enzyme activities and soil pH, as well as microbial biomass C (MBC) and soil pH, across different N addition rates and ratios.

The ratio of inorganic to organic N significantly affected the soil pH in most cases (*P* < 0.05) with the exception of under a N rate of 20 g N m^−2^ at day 60 (*P* = 0.07, Fig. 6). For both N addition rates, the soil pH at day 60 was significantly lower than those at day 30 (*P* < 0.05). The interactive effects of the N addition rate with the incubation time on the soil pH were statistically significant (*P* < 0.001). For each N ratio treatment, the N addition rate induced significant differences in the soil pH at day 60 (*P* < 0.05), but not at day 30 (*P* = 0.43). The soil pH was positively related to the βG activity and was negatively correlated with the βX activity, NAG activity and MBC (*P* < 0.05, Fig. 7). There were no correlations between the soil pH and αG, CBH, LAP and ALP (*P* = 0.31; 0.09; 0.38; 0.50).

## Discussion

### N addition stimulates hydrolytic enzyme activities

Based on the resource allocation theory^27^, N addition can increase the C and P acquisition enzyme activities and suppress enzyme activities associated with microbial N acquisition. Our result showed that N addition enhanced the enzyme activities associated with C and P mineralization, which is consistent with previous studies^2,27,28^. A meta-analysis based on 65 published studies revealed that N fertilization significantly increased the CBH, βX, βG, αG, and acid phosphatase activities by 6.4%, 11.0%, 11.2%, 12.0%, and 10.6%^2^.

However, N addition also enhanced the enzyme activities associated with microbial N acquisition in this study, which was unexpected. Some hypotheses could explain this response. First, enzyme activities are linked to nutrient availability^24^. Edaphic properties regulate N retention and supply^2^ and further influence the response of N acquiring enzyme activities to N addition. The soil in this study area is Salic Solonetz, which is a low fertility soil. The ratios of the soil C:N and microbial biomass C:N in this field were 15.8 and 12.3, respectively, and were higher than those in grasslands (13.8 and 8.3, respectively) at the global scale^29^. Thus, the tight N demand may stimulate N acquisition in this ecosystem. Second, soil pH is a primary control of enzyme activities and has direct biochemical effects on the enzyme activities immobilized in the soil matrix^6,30^. For saline-alkaline soil, N addition induces a decrease in soil pH and may produce optimal conditions for enzyme activities. NAG activity was negatively correlated with soil pH (Fig. 7), which supports this hypothesis. Finally, the enzyme activities are tied to the stoichiometry of the microbial nutrient demand^31^. N is distributed among several classes of polymers as well as humic molecules, so N acquisition strategies are linked to the C-substrate preferences of particular taxa^30^. The C:N acquisition ratios were close to 1:1 both in the control (1.02) and N addition (0.98) treatments. Our results agree with those reported by Jian *et al*.^2^, who found that N fertilization had no significant effects on the ratio of C acquisition to N acquisition enzyme activities. This result could be because the linkages between nutrient availability to soil decay on the basis of microbial allocation of resources to extracellular enzyme production^32^. Microbial nutrient demand is determined by the elemental stoichiometry of microbial biomass in relation to environmental nutrient availability^33^. However, the obtained data did not allow us to explore the relationships between enzyme activities and the stoichiometry of the microbial biomass and nutrient availability. The mineral N and microbial biomass N should be measured in further research to identify the underlying mechanisms of the response of enzyme activities to N addition.

### Effects of different inorganic to organic N ratios

Enzyme activities exhibited various responses to different inorganic to organic N ratios. The enzyme activities under mixed N addition were higher than those under single inorganic N addition, and the M treatment (inorganic N:organic N = 3:7) lead to the highest activities in most cases, which was consistent with the results of previous studies^15,16^. This result may be attributed to the fact that mixed N addition alleviates soil N limitation and organic nitrogen components, as glycine can supply utilizable C sources for soil microorganisms^34^, which further stimulate enzyme activities. On the other hand, the organic N is considerably easier to be assimilated by soil microorganisms than inorganic N^18^. Therefore, mixed N addition treatments, especially high organic N ratio treatments, are associated with better promotion of microbial activities^35^.

Furthermore, these results can be explained by several other reasons. First, although single N addition alleviated the soil N limitation, the balance of oxidized and reduced N and/or inorganic and organic N was disturbed^15^. Thus, single inorganic N addition or excessive organic N addition had low stimulating effects on soil enzyme activities compared to the M treatments. Second, different soil microorganisms may prefer different N forms (organic N or inorganic N), and as such, all of them are simultaneously stimulated by mixed N addition treatments^36^. It has been observed that organic N contributes 33% of the total N deposition at present in China^14^, which is equivalent to inorganic N:organic N = 7:3. Even if this ratio is constant, the increasing atmospheric nitrogen (N) deposition will stimulate biochemical cycles of soil nutrient elements such as C, N and P. If the organic N contribution to the total N deposition increases, this stimulating effect may be more remarkable.

### Effects of N addition rate

The response of soil enzyme activities to N addition not only vary with inorganic to organic N ratios, but are also influenced by N addition rate^37,38^. In our case, the enzyme activities involved in C cycling at the 20 g N m^−2^ rate were higher than those of the treatments at the 10 g N m^−2^ rate at day 30; whereas at day 60, the enzyme activities were greater at the 10 g N m^−2^ rate than at the 20 g N m^−2^ rate. The total inorganic N addition (T) treatment at the 20 g N m^−2^ rate at day 60 depressed the activities of CBH and βG compared to the control treatment. These results suggest that a stress condition may occur for N addition treatments at a high rate compared with the control treatment with the increasing N doses.

Some research has shown that a decrease in soil pH under high N addition has a deleterious impact on soil microbial communities^39^. Although the soil pH decreased with the increase of N addition rate and incubation time in this study, significant negatively relationships between the soil pH and MBC, βX activity and NAG activity were found, which are inconsistent with other studies^39^. It is possible that soil microorganisms and enzyme activities may be suppressed in untreated saline-alkaline soil with a high pH value (8.60 ± 0.01); thus, the decreases in soil pH could produce beneficial effects on soil microorganisms. The fact that the stimulating effect of N addition at a high rate on soil microorganisms and enzyme activities gradually weakened in this case could not be attributed to the decrease in soil pH. Dalmonech *et al*.^24^ also found that N addition greatly stimulated microbial enzyme activities in the first month, whereas the stimulating effects decreased with the increase of N addition rate. The reason for this observation could be that the slow increase of mineral N availability impairs the physiological status and hence inhibits the functionality of microorganism groups in all treated soils^24^.

### Normalization of activity levels

Normalizing activity levels to MBC allows for quantification of the amount of enzyme activities per unit MBC, which may be a qualitative metric of the microbial community function in response to specific treatments or conditions^20,40,41^. In fact, the MBC differences may account for the variability in enzyme activities among sites or treatments^30^. Thus, normalizing activity levels to MBC can help to eliminate the impact of spatial variability in MBC between samples. Our result showed that the variation pattern of enzyme activities normalized to soil dry mass across different N addition rates and ratios were similar to that of enzyme activities normalized to MBC (Fig. 4; Figs S1–3), which may be attributed to the fact that the collected soil sample was mixed to obtain a composite sample and was then allocated into different incubation bottles, which reduced the differences in soil samples among the treatments. On the other hand, although MBC significantly differed among the N ratio treatments, the absolute variation in MBC among the treatments was small and the coefficient of variation of MBC among the N ratio treatments ranged from 5% in 10 g N m^−2^ at day 30 to 6% in 10 g N m^−2^ at day 60. These findings indicate that normalizing activity levels to soil dry mass was appropriate in this study.

### The shortcomings of this study and prospects

Although the obtained results are robust and valuable, our understanding of the response of soil enzymatic activities to N addition at different inorganic to organic N ratios, the N addition rate and their interaction is still far from completion. On the one hand, N addition-induced shifts in the microbial community composition should yield corresponding shifts in the functional and metabolic potentials of the communities, resulting in a change in enzyme activities^13,23^. Identifying the change of the microbial community composition could be helpful to deeply understand the response of enzyme activities to N addition. On the other hand, plants may affect the response of soil enzymatic activities to N addition. N addition usually increases above-ground plant production, with corresponding changes in plant community composition, and decreases plant diversity and influences on N and C substrates, which will subsequently affect enzyme activities^9,42,43^. Future studies are needed to explore the response of enzyme activities to N addition under more complex field conditions.

## Conclusions

Our soil incubation experiment showed that seven hydrolytic enzyme activities involved in C, N and P cycling exhibited various responses to different inorganic to organic N ratios. The enzyme activities under the mixed N addition were higher than those under the single inorganic N addition and the M treatments (inorganic N:organic N = 3:7) provide the best combination for stimulating enzyme activities in most cases. Our results highlight the type and ratio of N are important factors in controlling soil enzyme activities. The response of soil enzyme activities to N addition also varied with the N addition rate. These findings enable us to better understand the effect of atmospheric N deposition on microbial activities in saline-alkaline soil. Furthermore, the microbial community composition and mineral N should be measured in further research to identify the underlying mechanisms of the response of enzyme activities to different inorganic to organic N ratios and N addition rate. The results of the laboratory experiment should be tested to determine if they are consistent with field condition.

## Materials and Methods

### Site description

The study site was conducted at the Songnen Grassland Ecology Research Station (44°45′N, 123°45′E) at the Changling Horse Breeding Farm, Jilin Province, northeastern China. The study area has a semiarid, continental climate. The mean annual temperature is 6.4 °C and the mean annual rainfall is 471 mm (1950–2004). Summer (June to August) precipitation accounts for more than 70% of the annual rainfall^44^. The frost-free period is 150 days. The soil in the study area is a saline-alkaline soil, which is equivalent to an Aqui-Alkalic Halosol based on the Chinese soil taxonomic system or a Salic Solonetz based on the WRB soil taxonomic system. The pH of the soil is between 8.0 and 9.0, the soil organic carbon content is 2.0%, and soil total nitrogen is 0.15%^45^. The vegetation is primarily composed of *L. chinensis*, *Phragmites australis*, *Chloris virgata*, and *Kalimeris integrifolia*^46^.

### Experimental design

Soil was collected from the top layers (0–15 cm) of a saline-alkaline *L. chinensis* meadow steppe in April 2016. The soil was sieved using a 2 mm mesh stainless steel screen, and then kept in a refrigerator at 4 °C prior to incubation. For the incubation experiment, 120 g soil was added to each incubation bottle (8.5 cm inside diameter × 10 cm height), with holes in the lid to ensure a supply of fresh air. The experimental design was full factorial, with two rates of N addition (10 g N m^−2^ and 20 g N m^−2^ during the 60 day incubation period) and four ratios of N addition, including total inorganic N (T, inorganic N:organic N = 10:0), low organic N (L, inorganic N:organic N = 7:3), medium organic N (M, inorganic N:organic N = 3:7), high organic N (H, inorganic N:organic N = 1:9). In this experiment, NH_4_NO_3_ was chosen as the inorganic N source. Based on the composition and sources of atmospheric organic N^14^, urea and glycine were chosen and mixed equally as the organic N source. N sources (as water solution) were sprayed once every 3 days. For the control treatment (CK), deionized water without N was added. There were eight incubation bottles per treatment.

To maintain microbial activity, glucose was added at 60 mg to each sample once every 3 days after applying N fertilizer, which is equivalent to 10 mg glucose g^−1^ dry soil during the whole incubation period (60 days). All samples were incubated for 60 days in a dark incubator at 25 °C and at a 60% water-holding capacity. Soil moisture was adjusted once per week with deionized water to maintain a constant moisture level. Four sample replicates per treatment were harvested to determine the MBC, pH values and enzymatic activities at days 30 and 60.

### Microbial biomass C, soil pH and enzymatic assays

MBC was determined following the chloroform fumigation-extraction method^47^. MBC was calculated according to the following formula: MBC = (organic C in fumigated extracts - organic C in unfumigated extracts)/*K*_E_, where *K*_E_ = 0.38. C in fumigated and unfumigated samples was determined with a TOC Analyzer (vario TOC, Elementar, Germany). Soil pH was measured using a PHS-3E glass pH electrode (Leichi Inc., China).

The activities of α-1,4-glucosidase (αG), β-1,4-xylosidase (βX), cellobiohydrolase (CBH), β-1,4-glucosidase (βG), L-leucine aminopeptidase (LAP), β-1,4-N-acetylglucosaminidase (NAG), and alkaline phosphatase (ALP) involved in decomposition or cycling of organic C, N or P were assayed using the method described by DeForest^48^. These enzyme activities were measured using fluorogenically labeled substrates, i.e., 4-MUB-α-_D_-glucopyranoside for αG, 4-MUB-β-_D_-xylopyranoside for βX, 4-MUB-β-_D_-cellobioside for CBH, 4-MUB-β-_D_-glucopyranoside for βG, L-leucine-7-amino-4-methylcoumarin for LAP, 4-MUB-N-acetyl-β-_D_-glucosaminide for NAG, and *p*-Nitrophenyl-phosphate for ALP (Table 2)^4,49^. Soil homogenates were prepared by adding 1 g of soil to 100 ml of 50 mM buffer with the pH adjusted to soil samples (pH ± 0.5). Sample homogenate (200 µl) was added, along with 50 µl of substrate for each target enzyme, into black 96 well microplates. Homogenate control wells contained 50 µl of buffer and 200 µl of sample homogenate. Substrate control wells contained 50 µl of substrate solution and 200 µl of buffer. Reference standard wells contained 50 µl of standard and 200 µl of buffer. Quench wells contained 50 µl of standard and 200 µl of sample homogenate. There were 8 replicates for each sample homogenate, homogenate control, substrate control, reference standard, and quench. Hydrolytic enzyme assays were incubated at 20 °C for 4 h, and the reactions were terminated by adding 10 µl of 1.0 M NaOH to each well. Fluorescence was measured using a fluorescence plate reader (TECAN infinite F200, Tecan Group, Switzerland) with excitation at 365 nm and emission at 445 nm. The enzyme activity was calculated as follows^41^:

1 $${\rm{Activity}}({\rm{nmol}}\,{{\rm{g}}}^{-1}\,{{\rm{h}}}^{-1})=\frac{{\rm{Net}}\,{\rm{Fluorescence}}\times {\rm{Buffer}}\,{\rm{volume}}({\rm{ml}})}{{\rm{Emission}}\,{\rm{coefficient}}\times {\rm{Homogenate}}\,{\rm{Volume}}({\rm{ml}})\times {\rm{Time}}({\rm{h}})\times {\rm{Soil}}\,{\rm{mass}}({\rm{g}})}$$

where,

2 $${\rm{Net}}\,{\rm{Fluorescence}}=(\frac{{\rm{Assay}}\,-\,{\rm{Homogenate}}\,{\rm{Control}}}{{\rm{Quench}}\,{\rm{Coefficient}}})-{\rm{Substrate}}\,{\rm{Control}}$$

3 $${\rm{Emission}}\,{\rm{Coeff}}.\,({\rm{fluorescence}}\,{{\rm{nmol}}}^{-1})=\,\frac{{\rm{Standard}}\,{\rm{Fluorescence}}}{[\frac{{\rm{Standard}}\,{\rm{Concentration}}({\rm{nmol}})\times {\rm{Assay}}\,{\rm{Volume}}({\rm{ml}})}{{\rm{Volume}}\,{\rm{of}}\,{\rm{Standard}}({\rm{ml}})}]}$$

4 $${\rm{Quench}}\,{\rm{Coeff}}.\,=\frac{{\rm{Quench}}\,{\rm{Control}}-{\rm{Homogenate}}\,{\rm{Control}}}{{\rm{Stand}}\,{\rm{Fluorescence}}}$$

**Table 2:** Abbreviations, commission number (EC), functions and corresponding substrate of the seven hydrolytic enzymes.

Enzyme	Abbreviation	EC	Function	Substrate
α-1,4-glucosidase	αG	3.2.1.20	Starch degradation	4-MUB-α-_D_-glucopyranoside
β-1,4-xylosidase	βX	3.2.1.37	Hemicellulose degradation	4-MUB-β-_D_-xylopyranoside
Cellobiohydrolase	CBH	3.2.1.91	Cellulose degradation	4-MUB-β-_D_-cellobioside
β-1,4-glucosidase	βG	3.2.1.21	Cellulose degradation	4-MUB-β-_D_-glucopyranoside
L-leucine aminopeptidase	LAP	3.4.11.1	Peptide breakdown	L-leucine-7-amino-4-methylcoumarin
β-1,4-N-acetylglucosaminidase	NAG	3.1.6.1	Chitin degradation	4-MUB-N-acetyl-β-_D_-glucosaminide
Alkaline phosphatase	ALP	3.1.3.1	Mineralizes organic P into phosphate	*p*-Nitrophenyl-phosphate

The soil C:N acquisition ratio was calculated as:

5 $${\rm{C}}:{\rm{N}}\,{\rm{acquisition}}=\,\mathrm{ln}({\rm{\beta }}G):\,\mathrm{ln}({\rm{NAG}}+{\rm{LAP}})$$

### Statistical analyses

The variance of the homogeneity of data was tested using Levene’s test, and the normality of data was tested using the Kolmogorov-Smirnov test. Three-way ANOVA was employed to analyze the main effects of the N ratio, N addition rate, and incubation time and their interactions on enzyme activities, MBC and pH. Differences among treatments were considered significant if *P* < 0.05 and were compared using Tukey’s HSD multiple comparison post hoc tests. The relationships between enzyme activities and MBC and pH were examined by linear regression analysis. Statistical analyses were performed with the SPSS 18.0 software (SPSS Inc., Chicago, USA). Graphs were created using Sigma Plot 12.5 software (Systat Software Inc., San Jose, USA).

## Electronic supplementary material

Supplementary information
