# Supplementary material for: Responses of hydrolytic enzyme activities in saline-alkaline soil to mixed inorganic and organic nitrogen addition
Source: Sci Rep. 2018 Mar 14;8:4543. doi: 10.1038/s41598-018-22813-9 (PMC5852051; doi:10.1038/s41598-018-22813-9)
Supplement: Supplementary file 1 — Supplementary information [file 41598_2018_22813_MOESM1_ESM.pdf]

Responses of hydrolytic enzyme activities in saline-alkaline soil to mixed inorganic and organic nitrogen addition

Baoku Shi[a], Junmei Zhang[a], Chengliang Wang[a], Jianying Ma[b], Wei Sun[a,*]

[a] Key Laboratory for Vegetation Ecology, Ministry of Education, Institute of Grassland Science, Northeast Normal University, Changchun 130024, China

[b] State Key Laboratory of Desert and Oasis Ecology, Xinjiang Institute of Ecology and Geography, Chinese Academy of Sciences, Urumqi 830011, China

* Corresponding author

Wei Sun

Tel.: +86 431 85098187

E-mail: sunwei@nenu.edu.cn

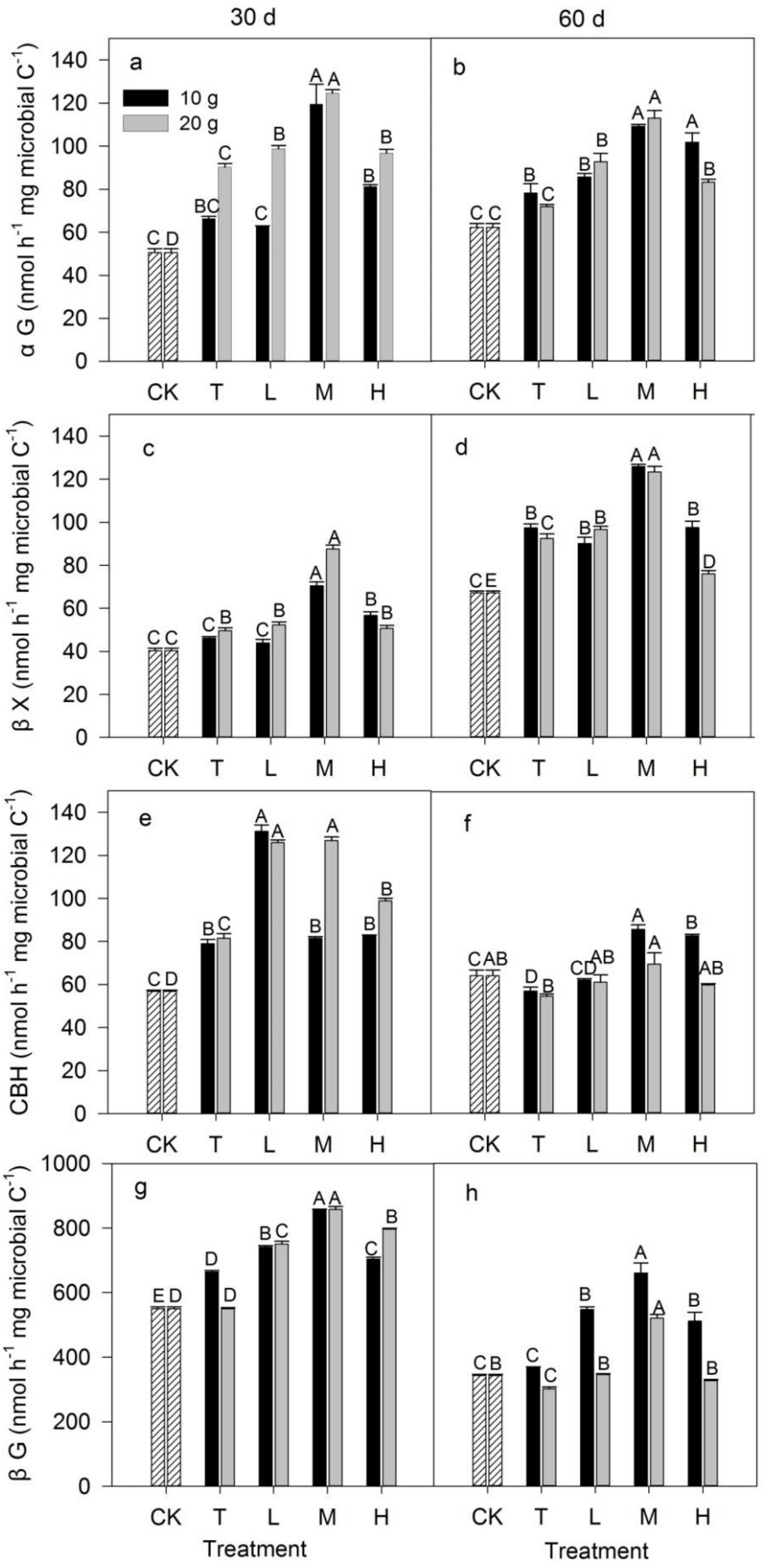

Figure S1 Response of soil enzyme activities per mass microbial carbon involved in C cycling to different inorganic to organic N ratios, different N rate (10 g N m^{-2} and 20 g N m^{-2}) and different incubation time (30 day and 60 day).

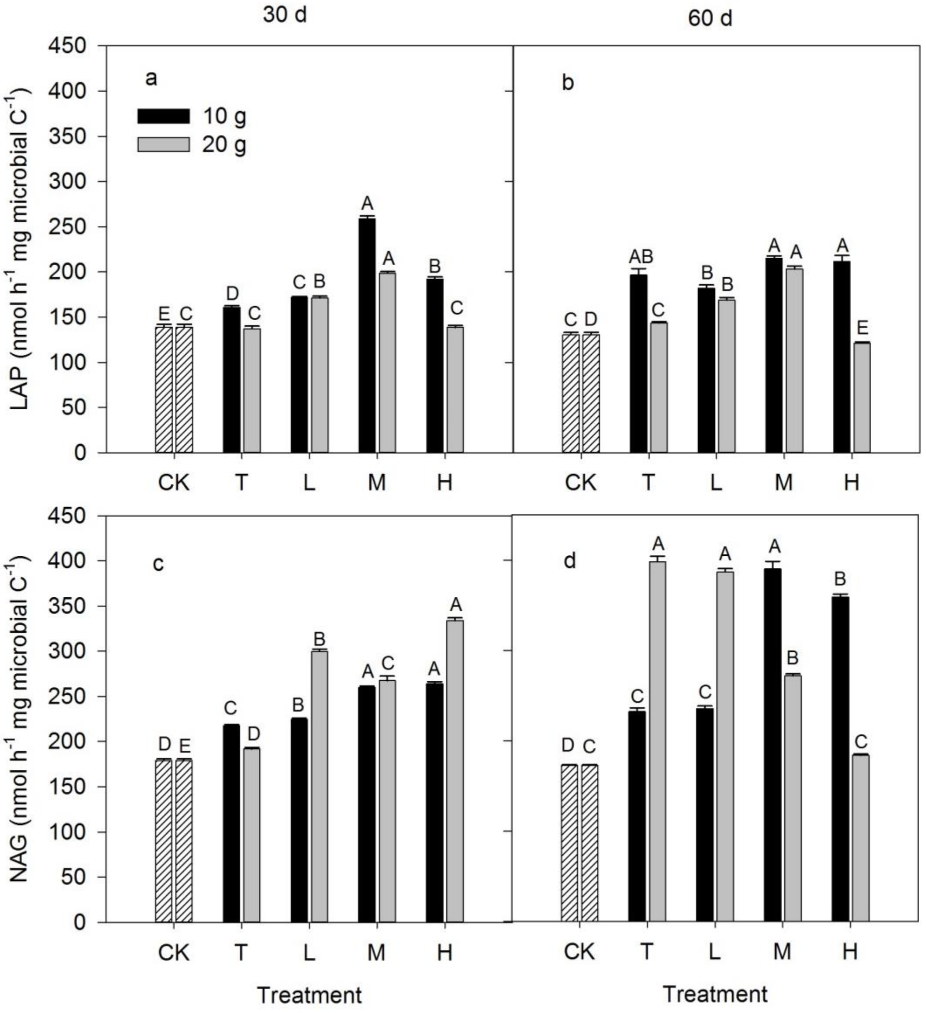

Figure S2 Response of soil enzyme activities per mass microbial carbon involved in N cycling to different inorganic to organic N ratios, different N rate (10 g N m^{-2} and 20 g N m^{-2}) and different incubation time (30 day and 60 day).

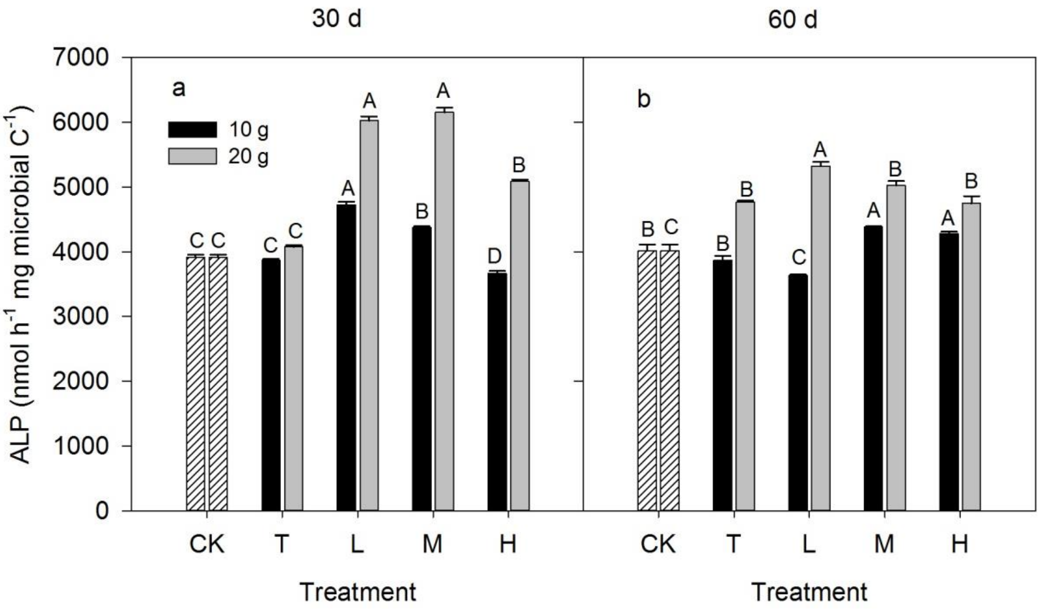

Figure S3 Response of soil enzyme activities per mass microbial carbon involved in P cycling to different inorganic to organic N ratios, different N rate (10 g N m^{-2} and 20 g N m^{-2}) and different incubation time (30 day and 60 day).
